# Traumatic mating by hand saw-like spines on the internal sac in *Pyrrhalta maculicollis* (Coleoptera, Chrysomelidae, Galerucinae)

**DOI:** 10.3897/zookeys.720.13015

**Published:** 2017-12-11

**Authors:** Yoko Matsumura, Haruki Suenaga, Yoshitaka Kamimura, Stanislav N. Gorb

**Affiliations:** 1 Department of Functional Morphology and Biomechanics, Zoological Institute, Kiel University, Am Botanischen Garten 1-9, D-24118 Kiel, Germany; 2 Sunshine A205, Nishiachi-chô 833-8, Kurashiki-shi, Okayama Pref., 710-0807, Japan; 3 Department of Biology, Keio University, 4-1-1 Hiyoshi, Yokohama 223-8521, Japan

**Keywords:** Copulation, genitalia, insect, internal sac, leaf beetles, mating systems

## Abstract

Morphology of the aedeagus and vagina of *Pyrrhalta maculicollis* and its closely related species were investigated. The internal sac of *P. maculicollis* bears hand saw-like spines, which are arranged in a row. Healing wounds were found on the vagina of this species, whose females were collected in the field during a reproductive season. However, the number of the wounds is low in comparison to the number of the spines. In addition, males of *P. tibialis* bear one spinous sclerite on the internal sac, but the female of this species show no wounds on the vagina. The vaginal wall is thicker in *P. maculicollis* and *P. tibialis* in comparison to other studied species, whose males bear no spinous sclerite. This thickening in *P. maculicollis* is hypothesized that they prevent damaging their own internal sac during everting and withdrawing the internal sac with the spines.

## Introduction

Traumatic mating is one of the well–observed phenomena in invertebrate mating systems (Lange et al. 2013, Reinhardt et al. 2015). Morphology of trauma-causing structures and ways of inflicting traumas diversified in the course of evolution (Lange et al. 2013). Examples of traumatic mating can be divided into three categories: traumatic insemination, traumatic secretion transfer, and traumatic penetration (Lange et al. 2013). In cases of the former two categories, wounds function as the entrance of sperm or seminal secretions without spermatozoa into the female body, respectively. There is continuing debate about the function and significance of mating trauma caused in the examples of the third category (Lange et al. 2013). On the contrary, female counter–adaptations, as response to traumatic mating, are less studied: however, some exemplary cases of female adaptions are known. For example, seed beetle females possess the thickened vaginal wall, as response to the spiny male penis (Rönn et al. 2007). Bed bug females have a spermalege that is an organ specialized to receive hypodermically injected sperm. Recent studies revealed that (1) physiological responses of this organ defend female body cavity against pathogens (Reinhardt et al. 2003) and that (2) the rubber–like protein, resilin, dominates in the wound inflicted areas of the organ to tolerate the traumatic cuticle penetration (Michels et al. 2015). Recently, additional examples of traumatic mating in a sea slug, earwig, and twisted wing parasite have been reported (Lange et al. 2014, Kamimura et al. 2016, Peinert et al. 2016). In one of the most mega-diversified group Coleoptera, hitherto only a few examples of traumatic mating are known: some Chrysomelidae (Bruchinae, Crudgington and Siva–Jothy 2000; Galerucinae, Flowers and Eberhard 2006), a species of Carabidae (Carabinae, Okuzaki et al. 2012), and as results of heterospecific mating in Carabidae (Sota and Kubota 1998).

In insects, male trauma inflicting structures usually locate in aedeagi, whose morphology is usually one of the best diagnoses especially in beetle systematics (Crowson 1981). Recently internal sac morphology is also described in many taxonomic papers. Possible wound inflicting structures, such as sharply pointed spines, have been described for many beetle groups, although it is usually unknown, whether they have a function in relation to traumatic mating. For example, regularly arranged spinous sclerites on the internal sac have been reported for the leaf beetles *Pyrrhalta maculicollis* and *P. sulcatipennis* (Nie et al. 2012, 2013). To examine the possibility of traumatic mating in species of this genus, we investigated morphology of the genitalia using species from Japan: *Pyrrhalta maculicollis*, *P. humeralis*, *P. tibialis*, and some members of the *Tricholochmaea semifulva* species complex (hereafter *Tricholochmaea semifulva* species complex) (Takizawa and Suenaga, unpubl. data). *Tricholochmaea semifulva* species complex was treated as *Pyrrhalta semifulva* before (Kimoto and Takizawa 1994) because of morphological affinity. Therefore *T. semifulva* species complex was chosen in the current paper for an outgroup comparison. Despite of a series of great works on *P. maculicollis* and related species on speciation (Nie et al. 2012, 2013, Zhang et al. 2014, 2015) and broad life history survey of Japanese leaf beetles (Takenaka 1963, Lee 1990, Kimoto and Takizawa 1994), we do not have much information on basic mating biology of *Pyrrhalta* species and applied a correlational method among species for the current study. First the morphology of male genitalia was investigated and then whether female vaginas have wounds or not to test correlations between spinous structures and wounds existences. Possible counter–adaptations in the females were also examined by measuring thickness of the vagina. In addition, irrespective of the functional significance of traumatic mating, wound–inflicting organs might require related adaptations in the male morphology, although this perspective has been totally overlooked in previous studies. In male beetles with a wound inflictor on the internal sac of the aedeagus, it may potentially harm the internal sac surface during repeated eversion and withdrawal of the internal sac. Based on comparisons among related species with an outgroup species, we also discuss possible male co–adaptations to traumatic mating.

## Materials and methods

The male and female genitalia of the following four species were examined: *Pyrrhalta maculicollis*, *P. humeralis*, *P. tibialis*, and *Tricholochmaea semifulva* species complex with a special focus on *P. maculicollis*, which has spines on the internal sac. In regards to the scientific name, *P. maculicollis* had been treated as *Xanthogaleruca maculicollis* previously (e.g. Beenen 2010). However, in the latest taxonomic paper (Nie et al. 2013), the genus *Xanthogaleruca* was treated as a synonym of the genus *Pyrrhalta* and this is followed here.

Examined specimens were mainly collected in the Okayama prefecture, Japan with some exceptions. For *P. maculicollis* we used individuals also from the Kanagawa prefecture, Japan, for *P. tibialis* two specimens from the Hokkaido prefecture, for *P. humeralis* one specimen from the Ehime prefecture, and for *T. semifulva* species complex two samples from the Oita prefecture.

To show general morphology and measure body sizes and the dimensions of genital spines, we dissected and observed samples under the stereomicroscopes (Nikon SMZ 745: Nikon Corporation, Tokyo, Japan; Olympus SZX12: Olympus Corporation, Tokyo, Japan; Leica M205 A with the camera Leica DFC420 and the software LAS 3.8: Leica Microscopy GmbH, Wetzlar, Germany) and the light microscope Zeiss Axioplan equipped with the camera Zeiss Axio Cam MRc (Carl Zeiss Microscopy GmbH, Jena, Germany). Then the sizes were measured with aids of the software Fiji (Schindelin et al. 2012) using the segmented line tool based on the taken images. For spine length measurement, we measured the length of five spines per male, one from each apical–, subapical–, middle–, subbasal–, and basal section of the spine row. For measurement of relatively well–sclerotized structures we used 99.5 % ethanol fixed specimens for *Pyrrhalta maculicollis* and dried specimens for other species. Potassium hydroxide was used to macerate muscles, when necessary, for visualization of skeletal structures.

To understand three-dimensional configuration of the aedeagus of *Pyrrhalta maculicollis* the aedeagus was dissected out from 99.5 % ethanol preserved specimens, dehydrated up to 100 %, and dried with a critical point drier (E3100 CPDA/Quorum Technologies LTD, Kent, UK). Then the sample was glued onto a thin–wall borosilicate glass capillary (120 × 1 mm, Hirschmann–Laborgeräte GmbH & Co. KG, Eberstadt, Germany) with super glue and scanned using the high–resolution micro–computed tomography (µCT) SkyScan 1172 (RJL Micro & Analytic GmbH, Karlsdorf–Neuthard, Germany) with a current of 250 µA and a voltage of 40 kV. Segmentation of each structure was carried out using the software Amira 5.4 (Visualization Sciences Group, Mérignac, France).

Some additional internal sac specimens of *Pyrrhalta maculicollis* were also dried with the same methods and sputter coated with gold–palladium (ca. 10 nm thickness) using the Leica EM SCD 500 High Vacuum Sputter Coater (Leica Microscopy GmbH) for detailed surface investigations using the Hitachi S4800 and TM3000 scanning electron microscopes (Hitachi High–Tech. Corp., Tokyo, Japan) at an accelerating voltage of 3 kV and ca. 15 kV, respectively. For interspecific comparisons, we also used internal sac samples dried at room temperature.

For interspecific comparisons of the vaginal wall thickness, we dissected female vaginas from freshly killed samples in phosphate–buffered saline (PBS; Carl Roth GmbH & Co. KG, Karlsruhe, Germany) and fixed with 2.5 % glutaraldehyde for one to three weeks. Two females per species were fixed except for *P. tibialis*, for which only one sample was treated. The samples were washed with PBS at least three times, dehydrated with a series of ethanol up to 100 % ethanol. Then they were gradually replaced with Epon 812 (Glycidether 100; Carl Roth GmbH & Co. KG), and finally the samples were embedded in the Epon resin. All procedures were processed at room temperature, but polymerisation was done at 60 °C for two days. Semi–thin sections (ca. 300–700 nm) were prepared using the Leica EM UC7 ultramicrotome (Leica Microscopy GmbH). Sections were stained with 0.1 % toluidine blue for three to four hours, and overstained dye was removed by retaining the slices in glycerine for two days. Images of the sections were then taken with the light microscope Zeiss Axioplan equipped with the camera Zeiss Axio Cam MRc. Following the method of Rönn et al. (2007), the areas of muscles and epidermis plus cuticle were measured with aids of the software Fiji.

A Fisher’s exact probability test was adopted for comparing the occurrence rate of mating trauma among species. All statistical analyses were carried out using R 3.2.0 (R Core Team 2015).

## Results

### Anatomy of the male aedeagus of *Pyrrhalta maculicollis*

**Figures 1–5.**
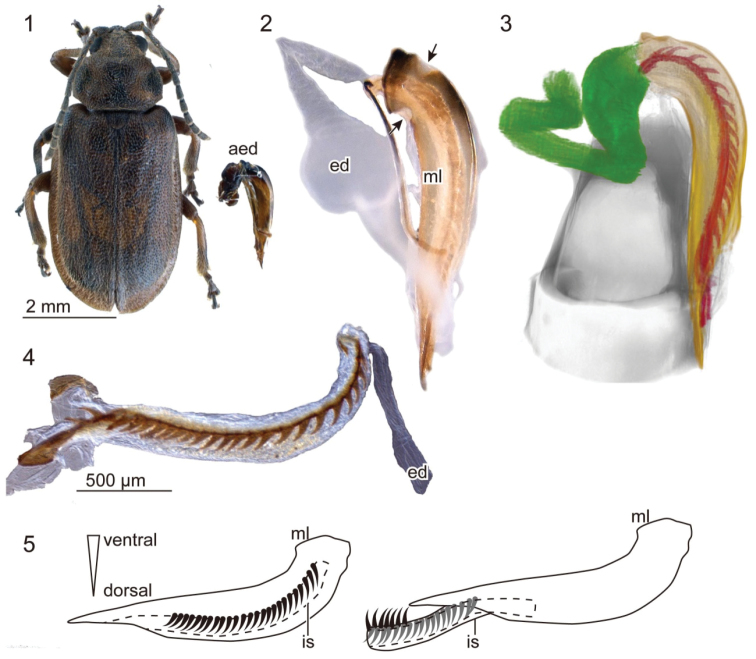
Aedeagus of *Pyrrhalta maculicollis* in the lateral view **1** relative size of the aedeagus compared to body size **2** the aedeagus and a part of the ejaculatory duct, muscles completely macerated. Two arrows point to the membranous areas of the median lobe **3** a micro CT scanned and segmented aedeagus and a part of the ejaculatory duct. The green structure represents the part of the ejaculatory duct with well–developed circular muscles, the yellow one represents the median lobe, and the red ones represent spines on the internal sac **4** the internal sac at rest with a part of the ejaculatory duct (ed) **5** schemes of the aedeagus at rest (left) and with partly evaginated internal sac during copulation (right). Abbreviations: aed, aedeagus; ed, ejaculatory duct; is, internal sac; ml, median lobe.

**Figures 6–8.**
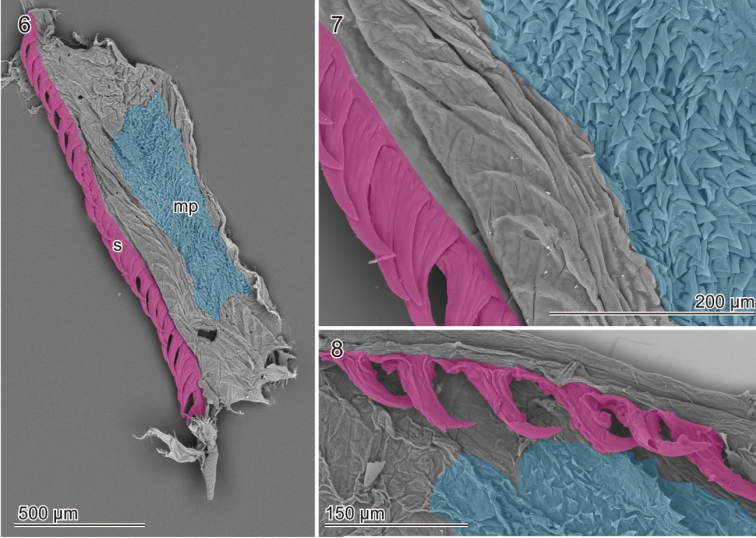
The internal sac membrane and spines of *Pyrrhalta maculicollis*. **6, 7** the sample was cut laterally, opened, dehydrated, and dried at the critical point **8** the sample dried at room temperature. The surfaces of the spines is slightly shrunken in comparison to that depicted in 7. Abbreviations: mp, membranous projections; s, spines; each is highlighted with blue and pink, respectively.

The males have a relatively stout aedeagus (Fig. 1). The median lobe has two small membranous regions on the proximal part in the lateral view (Fig. 2, arrows). The distal part of the ejaculatory duct, which is located adjacent the aedeagus, is enclosed by well–developed circular muscles (Fig. 3). This section of the ejaculatory duct is widened and its wall is folded at repose. When the surrounding muscles are macerated, this section is swollen (Fig. 2). Well–developed spines are situated on the ventral side of the internal sac, and the spines are caudally curved (Figs 3, 4). This means that the evaginated internal sac wears the spines pointing in the opposite direction (Fig. 5), which will result in anchoring the vagina during copulation (Figs 5, 13). The spines are pigmented and look like well-sclerotized structures. However, in the samples that had been dried at room temperature, the surface of the spines is slightly shrunk (Fig. 8) compared with that of critical-point dried samples (Figs 6, 7). The number and size of spines are variable among individuals within a population (Kanagawa, Japan; Table 1). The surface of the internal sac is largely covered with tiny projections (Figs 6, 7).

**Table 1.** The measurements of the male spines in *Pyrrhalta maculicollis*, Kanagawa population in Japan.

	**N**	**Mean ± S.D. (min.–max.)**
Body size (mm)	6	6.00 ± 0.12 (5.84–6.11)
Spine number	7	23.6 ± 2.4 (20– 8)
Spine size (μm)	7	126.2 ± 10.7 (110.8–145.0)
Internal sac length (μm)	7	2305 ± 83.9 (2204–2432)

### Copulatory wounds in female *Pyrrhalta maculicollis*

**Figures 9–12.**
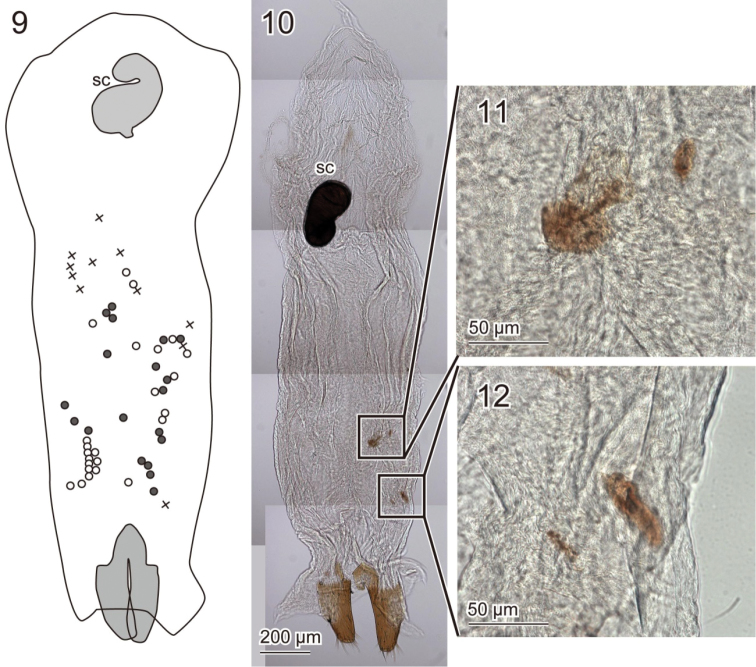
Wounds observed on the vagina of *Pyrrhalta maculicollis* **9** schemes of three female vaginas, which had largest numbers of wounds, are shown. Each symbol represents one individual **10** one representative with four wounds **11, 12** enlarged images of the wounds. Abbreviations: sc: spermathecal capsule.

The vaginas of 13 female *Pyrrhalta maculicollis* were examined, collected during the reproductive season in the field (Kanagawa, Japan) and probably had already copulated. In most females (*N* = 11), 11.8 ± 6.3 (mean ± SD) wounds (2–25) were detected on both the ventral and dorsal sides of the vaginal wall, whereas no wound was found in the other two females. The sizes of wounds varied considerably and some were large enough to be visible under the stereomicroscope (Figs 10–12). Contrary to the male spines, the wounds were never aligned in a straight line (Figs 9–12). All wounds in females were observed in the caudal area posterior to the spermathecal capsule (Figs 9–12).

### General morphology of male aedeagus in related species

The shape of the median lobe of the studied species is similar except for *Tricholochmaea semifulva* species complex, whose median lobe is relatively slender.

**Figures 13–16.**
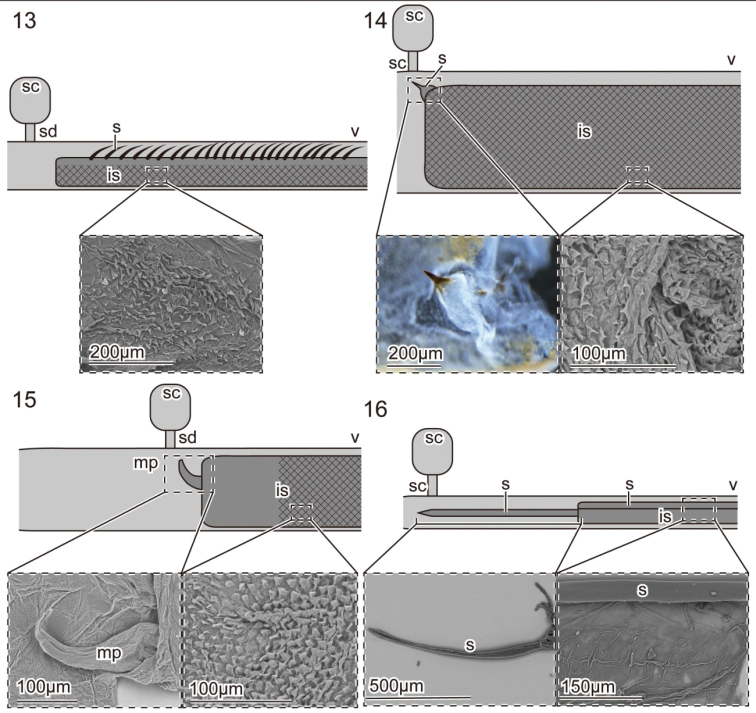
Schemes of hypothetical couplings between male and female genitalia, light microscopy (LM) and scanning electron microscopy (SEM) images of the male internal sac **13** *Pyrrhalta maculicollis* **14** *P. tibialis* **15** *P. humeralis* **16** *Tricholochmaea semifulva* species complex. The schemes were created using information on morphology and dimensions of both male and female genitalia, but the genital coupling has not been experimentally proven. The meshed areas in the male internal sacs show areas covered by tiny projections, as shown in the SEM images. All samples were dried at room temperature. Abbreviations: is, internal sac; mp, membranous projection; s, sclerite; sc, spermathecal capsule; sd, spermathecal duct; v, vagina.

The internal sac of *Pyrrhalta tibialis* and *P. humeralis* is broader than that of *P. maculicollis* and *Tricholochmaea semifulva* species complex (Figs 13–16). In *P. tibialis*, a sclerotized and spinous sclerite is present near the orifice of the ejaculatory duct (Fig. 14). The surface is almost completely and densely covered with tiny membranous projections (Fig. 14). Remarkable sclerites are absent in *P. humeralis*, but membranous projections cover the proximal half of the internal sac, and a membranous rod is present on the tip of the internal sac (Fig. 15). Two long sclerites, as long as the internal sac, are situated on the tip of the internal sac and along the internal sac in *Tricholochmaea semifulva* species complex (Fig. 16). Membranous projections found in *Pyrrhalta* species are absent on the internal sac surface of *T. semifulva* species complex.

### Vagina wall comparison among species

**Figures 17–25.**
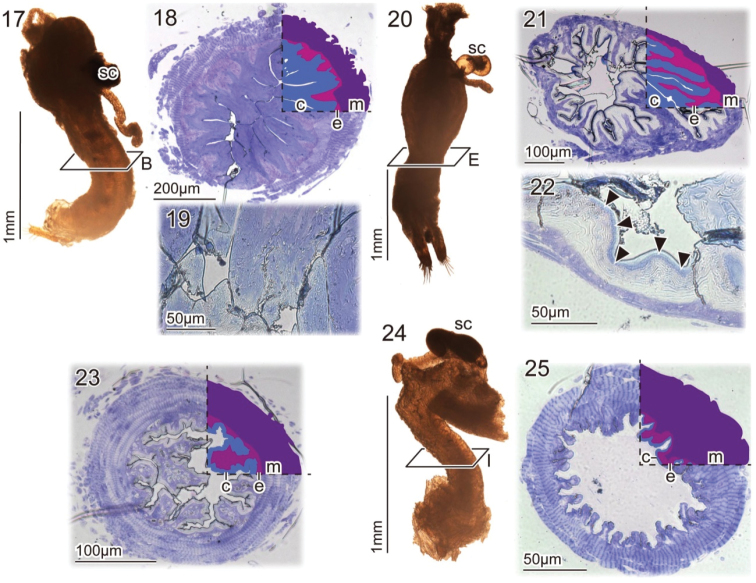
Female reproductive systems: **17–19** *Pyrrhalta maculicollis* **20–22** *P. tibialis* **23** *P. humeralis* **24–25** *Tricholochmaea semifulva* species complex. **18, 21, 23, 25** show the cross sections of the lower part of the vagina, where wounds were found in *P. maculicollis*; each cuticular, epidermal, and muscular layer are partly highlighted in the upper right corner. **19, 22** show enlarged parts of the cuticular, epidermal, and muscular layers; these samples were embedded in glycerine for two days before taking the images. Arrowheads in **22** point to the strip stained with toluidine blue. Abbreviations: c, cuticle; e, epidermis; m, muscles; sc, spermathecal capsule.

The vaginal wall of *Pyrrhalta maculicollis* and *P. tibialis* shows relatively well developed epidermis and cuticle layers, if compared to that of the other species (Figs 17–22), while muscles are well developed in *P. humeralis* and *Tricholochmaea semifulva* species complex (Figs 23–25). Proportions of the epidermis + cuticle areas to the area of the muscle layer are variable among species, and these values in *P. maculicollis* and *P. tibialis* are higher than those of the others: 1.76–1.85 (*N* = 2) in *P. maculicollis*, 2.22–5.81 (*N* = 2) in *P. tibialis*, 0.60 (*N* = 1) in *P. humeralis*, 0.27–0.62 (*N* = 2) in *T. semifulva* species complex (Table 2). The thick cuticular layers in *P. maculicollis* and *P. tibialis* are not strongly stained with toluidine blue. However staining patterns are different between the species, i.e. *P. maculicollis* cuticular layers are weakly and homogeneously stained (Fig. 19) and *P. tibialis* cuticular layers are not stained except for a stained stripe (Fig. 22, arrowheads).

**Table 2.** The measurements of the areas of the vagina muscles and epidermal plus cuticular layers.

**Species**	**N**	**Areas of muscles (µm^2^)**	**Areas of epidermis and cuticle (µm^2^)**
*Pyrrhalta maculicollis*	2	102545 / 45674	180528 / 84607
*Pyrrhalta tibialis*	2	12574 / 40076	73012 / 88799
*Pyrrhalta humeralis*	1	27599	16555
*Tricholochmaea semifulva* species complex	2	38635 / 13166	23952 / 3601

Any possible healing wounds were not found in the vagina of *P. tibialis* (*N* = 5) and *P. humeralis* (*N* = 4), indicating that mating trauma, if any, occurs significantly less frequently in these species than in *P. maculicollis* (Fisher’s exact probability test: *P* = 0.0025–0.0063). However, five small melanised patches were found in one out of four females of the *T. semifulva* species complex examined. Those patches were similar to the small ones in Figs 11, 12.

## Discussion

Among the examined *Pyrrhalta* spp., wounds were found significantly more in the vagina of *P. maculicollis*. This finding supports the view that the hand saw-like spines of the internal sac, which is characteristic of this species, are responsible for traumatic mating. Although we have neither compared virgin females with mated ones nor examined the genital coupling of the species, it is reasonable to estimate that the regularly arranged male spines are everted and face to the vaginal wall during copulations (Fig. 13). The everted spines must be arranged like anchoring to the vaginal wall (Fig. 13). However, the number of the wounds of the females, collected from the field, was less in comparison to the number of the spinous sclerites of the internal sac, and the traces of the wounds do not coincide with the male spine arrangement, as typically seen in ants with a similar hand saw-like spines (Kamimura 2007). This would mean that the spines of *P. maculicollis* are not stiff enough to always inflict wounds on the female vagina. In accordance with this view, the air-dried spines shrunk in comparison to the spines dried at the critical point. The spine surface can be less stiff than the spine internal part. Moreover, we found that the *P. tibialis* also has a plausible wound inflictor in the internal sac. On the contrary to *P. maculicollis*, however, we failed to detect wounds on five observed vaginas of *P. tibialis*. Additional studies are necessary to elucidate functions of the single spinous sclerite in *P. tibialis*.

As in the cases of seed beetles (Rönn et al. 2007), the high cuticular + epidermal layer area ratio in comparison to the muscle layer area in *P. maculicollis* vagina likely represents a counter–adaptation to traumatic mating, although we have not statistically analysed our data due to the small sample sizes. The relatively high cuticular + epidermal layer ratio was found also in *P. tibialis*, although it has not been confirmed that the spinous sclerite inflicts traumas during copulation in *P. tibialis*. It is conceivable that the material composition of the thickened cuticular layers is different between *P. maculicollis* and *P. tibialis*, because the staining of the cuticle layers differs between the species. Since toluidine blue strongly stains the rubber–like protein, resilin, which was demonstrated to enhance female tolerance against traumatic mating in bed bugs (Michels et al. 2015, 2016), we expected the highest stainability by toluidine blue in the vaginas of *P. maculicollis* among the species examined. However, resilin unlikely distributes much in the cuticle layers of *P. maculicollis* and the related species due to their low stainability by toluidine blue (Figs 19, 22). Thickening the vaginal wall alone is probably sufficient to be a counter–adaption against the spinous sclerites on the internal sac in *P. maculicollis*. Judged from the observed thin cuticular layers in the vagina of *T. semifulva* species complex, the thick vaginal cuticular layers likely represent a derived state in the genus *Pyrrhalta*. For estimating the origin of the female vaginal wall thickening, phylogenetic hypotheses of the relationship among different *Pyrrhalta* species should be developed in future studies.

The internal sac of *P. maculicollis* is relatively narrow in comparison to other *Pyrrhalta* species. The narrow internal sac would be problematic for bearing the spiny sclerites, since the spines may harm the internal sac, especially its dorsal surface, during its eversion and withdrawal (Fig. 5). It can be hypothesized that the presence of tiny and densely covering projections on the male internal sac presumably aid in avoiding such self–harming, and therefore represent possible male co–adaptation for traumatic mating. However, the tiny projections had likely evolved in *Pyrrhalta* and were found in all examined species of the genus. Accordingly, they must have other presently unknown functions, which have been preadaptations for the evolution of traumatic mating. The species rich galerucine genus *Pyrrhalta* contains more than 110 described species (Xue and Yang 2010, Nie et al. 2013), for which phylogenetic relationships at the species level are unknown. The morphology of the internal sac/vagina and the phylogeny of this group must be investigated comprehensively in future studies, for better understanding the genital evolution of this group.

In comparison to the seed beetles, whose spinous sclerites on the internal sac are arranged three dimensionally (Crudgington and Siva–Jothy 2000), the spines of *P. maculicollis*, which are arranged rather two–dimensionally, can be easily counted. Moreover, as shown in the present study, the spine number in this beetle is highly variable even within a population (Table 1). As well as possible variations among populations (see Dougherty et al. 2017 for a case of the seed beetle), it can cause mismatches between male and female genitalia. Some combinations may more severely harm females than others, enabling us to detect (1) female costs and (2) female counter adaptations. Despite that this species can potentially be a model system for future experimental studies of traumatic mating, hitherto no comprehensive study has been published for the mating biology of the *Pyrrhalta* spp. Establishment of rearing techniques are warranted especially for *P. maculicollis* to confirm our predictions on the trauma-causing functions of the genital spines with direct evidence.
